# Topical brimonidine induced acute uveal effusion in a patient with nanophthalmos: a case report

**DOI:** 10.1186/s12886-023-03107-9

**Published:** 2023-10-09

**Authors:** Yakun Li, Qi Zhou

**Affiliations:** 1 Department of Ophthalmology, Peking Union Medical College, Peking Union Medical College Hospital, Chinese Academy of Medical Sciences, Shuai Fu Yuan, Dongcheng District, Beijing, 100730 China; 2 Department of Ophthalmology, The Second Affiliated Hospital of Hebei North University, Zhangjiakou, 075100 China

**Keywords:** Acute uveal effusion, Topical brimonidine, Nanophthalmos

## Abstract

**Background:**

We report a case of uveal effusion in a nanophthalmic eye after topical use of brimonidine.

**Case presentation:**

A 42-year-old male patient with nanophthalmos experienced sudden blurred vision in the right eye after using topical brimonidine when picking up tennis balls repeatedly 6 weeks after bilateral YAG peripheral iridotomy. Ocular examination showed wide choroidal and exudative retinal detachment in the temporal and inferior region, involving the macula. Acute uveal effusion in the right, bilateral nanophthalmos was diagnosed. Oral and topical corticosteroids, combined with topical nonsteroids and atropine led to a complete resolution of the uveal effusion after one month.

**Conclusion:**

This case suggested a possible causal relationship between the topical use of brimonidine and acute uveal effusion in patients with nanophthalmos. Topical brimonidine should be used with caution in nanophthalmic eyes.

## Background

Uveal effusion syndrome (UES) is a rare eye disease that seriously affects visual function. It is characterized by the accumulation of fluid in the suprachoroidal space, with subsequent choroidal and retinal detachment. This disease is most common in patients with nanophthalmos, especially in middle-aged men, but can also occur in normal eyes [1]. Nanophthalmos is a congenital developmental abnormality characterized by high hyperopia (+ 12.00 ~ + 19.00D), short axial length (less than 20 mm), small corneal diameter (less than 10 mm), abnormal scleral thickening, optic nerve head crowding, and venous congestion. Scleral thickening with collagen abnormality in this extremely small eye was supposed to be the cause of uveal effusion [2]. Uveal effusion induced by laser, surgery, and trauma has been reported. This is the first report of uveal effusion that may have been induced by topical brimonidine administration.

## Case presentation

**Fig. 1:**
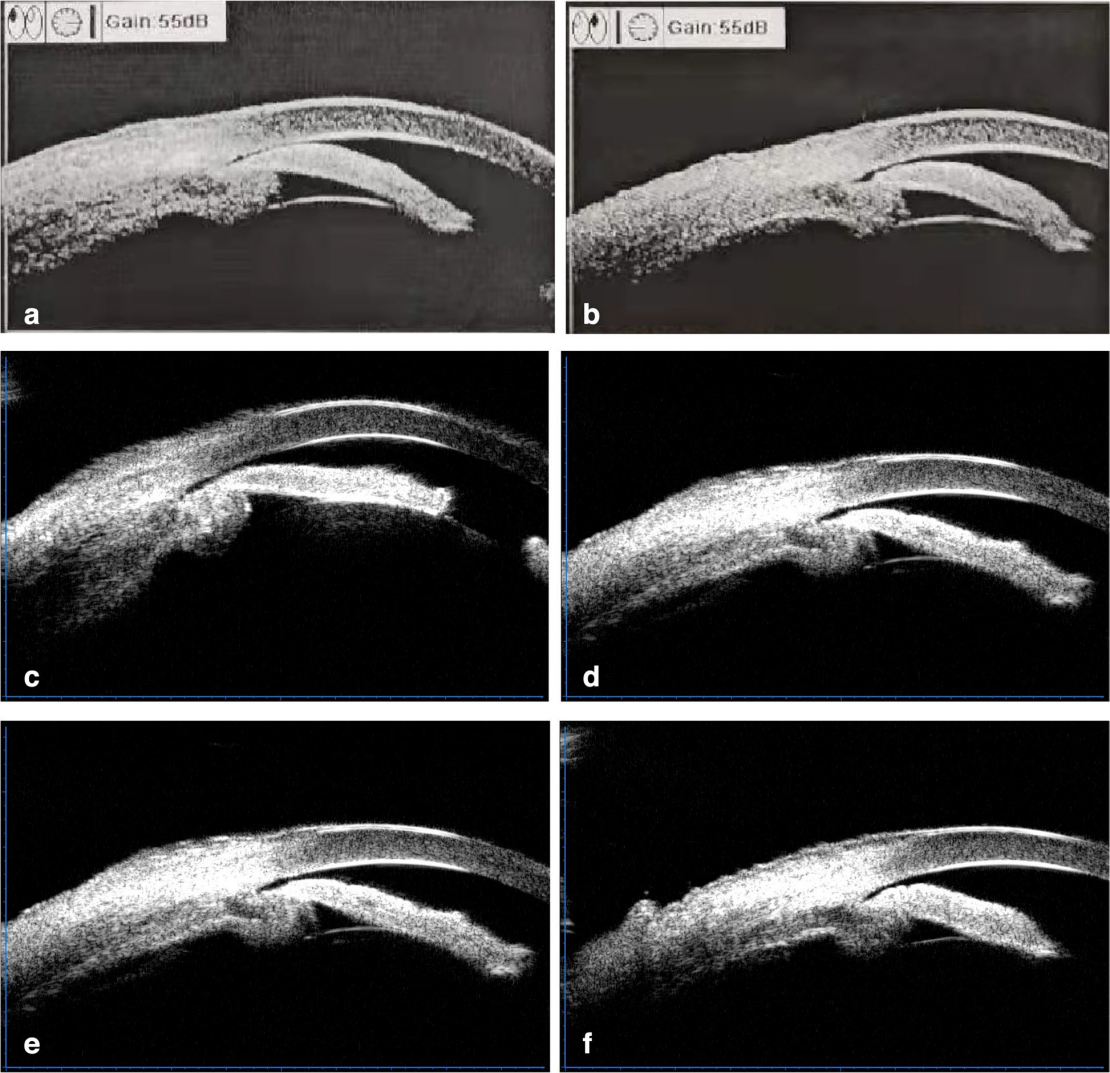
UBM results of the right (left side) and left (right side) eye. **a**, **b** UBM before YAGlaser showed a shallow anterior chamber with a thick ciliary body and partial superficial detachment in both eyes. **c**, **d** UBM after YAG laser showed a thick ciliary body and partial superficial detachment in both eyes similar to that before laser. **e**, **f** UBM 1 month after acute uveal effusion showed a complete resolution of the edema and the detachment of the ciliary body

**Fig. 2:**
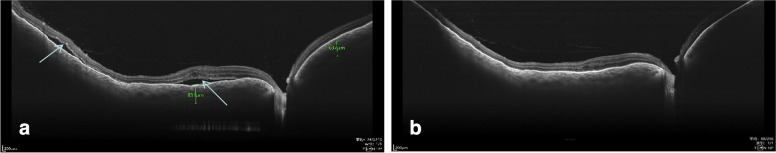
OCT of the macular area of the right eye. **a** OCT showed fluid accumulation (arrow) beneath the neurosensory retina, resulting in mild serous detachment. **b** OCT 1 month after acute effusion showed the subretinal serous fluid was absorbed and the retinal detachment was reattached

**Fig. 3:**
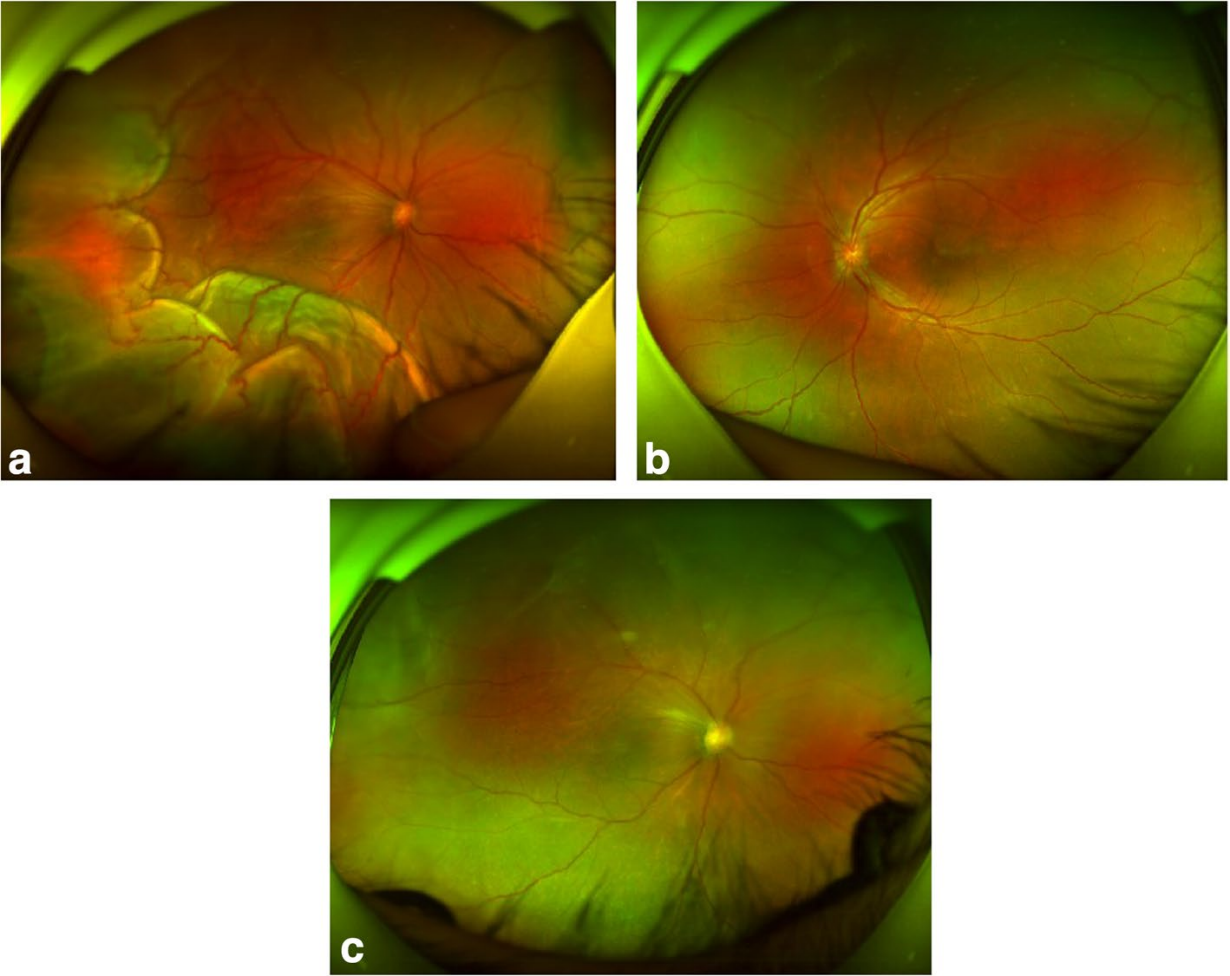
The wide-angle fundus photos of the right and left eye. **a** Fundus photo of the right eye showed a crowded optic disc, mild dilated retinal veins, and wide retinal and choroidal detachment in the temporal and inferior region with macular folds in the right eye. **b** Fundus photo of the left eye without the use of topical brimonidine. **c** Fundus photo of the right eye showed a complete reattachment of retinal and choroidal detachment 1 month after acute effusion

A 42-year-old man presented to the Department of Ophthalmology with a 6-day history of blurred vision in the right eye. He was physically healthy besides having high blood uric acid. His blood pressure was 125/80mmHg. He had intermittent red eye and pain in both eyes in the past 3 years and was diagnosed to have primary angle closure (PAC) at another hospital. Ultrasound biomicroscopy (UBM) before YAG laser showed areas of ciliary body edema and partial superficial detachment in both eyes (Fig. 1). Nanophthalmos, as well as abnormal UBM findings happened to be overlooked by his previous physician. YAG laser peripheral iridotomy was done in both eyes 6 weeks ago without any pretreatment. Four weeks after the laser, no changes in visual acuity were found. His IOP was 24mmHg in the right eye, and 16mmHg in the left eye. UBM test showed no worsening of the ciliary body edema and detachment in both eyes compared with previous results (Fig. 1). No choroidal and retinal detachment was detected by the ultrasound B scan. Azopt (Brinzolamide eye drop,Alcon) was given in the hope to relive ciliary edema and fluid in the supraciliary space. One week later, a routine checkup was performed for the patient in another hospital, and he was given Alphagan (Brimonidine Tartrate Eye Drops,Allergan). After using Alphagan, he went to the tennis court to help with picking up balls for his daughter, and after the session, a sudden blurred vision happened. Choroidal and exudative retinal detachment in the temporal and inferior region involving the macula area was detected (Figs. 2 and 3). The results of the ophthalmic examination were listed in Table 1. The patient was diagnosed as having uveal effusion in the right, bilateral nanophthalmos, and bilateral cataract (mild).

**Table 1:** Summary of ophthalmic examinations

	Right eye	Left eye
BCVA	2/20 (+ 14.00 D)	10/20 (+ 14.00 D)
IOP	16mmHg	16mmHg
Corneal Diameter	10 mm	10 mm
Central Corneal Thickness	572 μm	570 μm
Axial Length	15.84 mm	15.64 mm
Anterior Chamber Depth	1.51 mm	1.62 mm
Peripheral anterior chamber	≤1/4 CT	≤ 1/4 CT
iris	open iris hole	open iris hole
Lens	mild opacity	mild opacity
Fundus (Fig. 2)	A/V = 2:3, slightly dilated veins, Choroidal and retinal detachment with macular involvement	A/V = 2:3, slightly dilated veins, the retina was in place
Optic Nerve	Crowed, with a pale temporal rim	Crowed optic disc
Macular	retinal folds	Normal
Retinochoroidal scleral (RCS) complex thickness	2.46 mm	2.62 mm

Conservative therapy was provided as follows: Terminate the use of Alphagan and use Azopt. Pred Forte (Prednisolone Acetate Ophthalmic Suspension,Allergn) od q1h, Pranopulin (Pranoprofen Eye Drops,Senju Pharmaceutical) od qid, os bid, DiShan(Atropine sulfate ophthalmic gel,Sinqi) od tid, and tapering dose of prednisolone acetate (Sine) 60 mg qd was given. The blurred vision gradually improved. The intraocular pressure was stable and the area of retinal and choroidal detachment was significantly reduced. One month later, UBM examination did not find signs of ciliary body edema or detachment in both eyes (Fig. 1). Wide-angle fundus photography showed complete reattachment of the choroid and retina with the resolution of the serous fluid in the right eye (Fig. 3). Macular OCT showed that the neuroepithelial detachment in the macular region of the right eye was greatly smoothed (Fig. 2). At the last visit before the formulation of this paper, the patient’s condition was stable and there was no recurrence.

## Discussion and conclusions

This is the first reported case of acute uveal effusion that happened after using topical brimonidine. The patient was nanophthalmic in both eyes without a history of high blood pressure or diabetes milieus. There was no hypertensive retinopathy or choroidopathy. He had asymptomatic mild idiopathic ciliary choroidal detachment before the YAG peripheral iridotomy. This condition did not worsen after laser. Nevertheless, an acute exudative retinal and choroidal detachment appeared in the eye after using topical brimonidine without obvious inflammation and IOP spike, after being in a squatting position repeatedly. Conservative therapy including steroids, brinzolamide, and atropine led to a complete recovery of choroidal and retinal detachment.

Several factors may have contributed to the uveal effusion in this case. Nanophthalmic eyes are prone to uveal effusion because of their thickened sclera and abnormality in scleral collagen. The thickened sclera compresses the vortex vein, impedes blood reflux, and leads to congestion of the choroidal veins. The abnormality in scleral collagen, like interfibrous glucosamine-like deposition [3], decreases scleral macromolecular permeability, affects the trans-scleral intraocular fluid outflow, and will accelerate the fluid accumulation in the supra-choroidal cavity [4]. The middle-aged male in our case has high hyperopia, small cornea, short AL, and increased retinochoroidal scleral (RCS) complex thickness. His clinical features were consistent with the diagnosis of nanophthalmos, and his UBM showed ciliary body edema and partial superficial detachment in both eyes before laser. MasanobuUyama showed that patients with nanophthalmos are in the subclinical stage of uveal leakage syndrome even when they are asymptomatic. Stimulations such as ocular surgery and laser therapy (YAG iridotomy or ALT) will lead to the aggravation of choroidal fluid [3, 5, 6]. In this case, laser treatment did not worsen the uveal condition. However, it might have put the patient in a riskier situation that any further stimulation would cause acute retinal and choroidal detachment. UES occurred after the patient frequently squatted to pick up tennis balls, and this maneuver might be a triggering stimulation. Studies have found increases in choroidal blood flow, ocular perfusion pressure, and vascular resistance during squatting [7]. This might aggravate the existing congestion of choroidal vessels in nanophthalmic eyes. While the cause of uveal effusion may have been multifactorial, acute UES occurred in the right eye treated with brimonidine, not the similar fellow eye.

Therefore, topical brimonidine usage could have been a possible cause in this case. Brimonidine is a selective alpha 2 adrenoceptor agonist used in the treatment of glaucoma and ocular hypertension. It is commonly used immediately after YAG laser peripheral iridotomy. It lowers IOP by reducing aqueous humor production and by increasing its outflow via the uveoscleral pathway. The peak IOP lowing effect of brimonidine takes place 2 h after instillation [8]. It can quickly pass the choroid-scleral when the permeability of RPE increases [9]. Brimonidine is also a very effective vasoconstrictor that can reduce nitric oxide-mediated vasodilation of retinal arterioles [10], and this effect is more obvious in arterioles smaller than 200 μm [11]. Even a single administration of topical alpha 2 agonists can cause choroidal contraction and blood flow changes [12]. The blood flow in the choroid is thought to be maintained by an autoregulation mechanism. When the ocular perfusion pressure and intraocular pressure fluctuate significantly, this autoregulation mechanism will be destroyed [13, 14]. Topical alpha 2 agonists have been found to decrease choroidal blood flow and increase vascular resistance during squatting [7]. In this case, the topical use of brimonidine contracted the choroidal vessels when the patient was in a squatting position. It might have damaged the autoregulation of choroidal microcirculation, causing acute uveal leakage and exudative retinal choroidal detachment.

Uveal effusion is traditionally treated first by conservative therapies. Carbonic anhydrase inhibitor has a positive clinical use in UES patients due to its edema-reducing effects and impacts on choroidal circulation [15]. Oral and topical steroids are commonly used to control inflammation and restore ocular barriers. Rapid resolution of recurrent exudative retinal detachment in a patient with nanophthalmos using NSAIDs has been reported [16]. Atropine is also widely used in cases with uveal leakage due to its effect on relaxing the ciliary muscle and resetting the ciliary body and choroid. Some cases that did not respond to those treatments or had side effects might require surgical treatment, like deep posterior sclerotomy. In our case, the exact mechanism by which the UES resolved was unclear. The termination of topical brimonidine might have prevented the progression of the disease. The use of oral and topical steroids combined with topical nonsteroid, atropine, and carbonic anhydrase inhibitor resulted in a complete resolution of exudative retinal and choroidal detachment.

To sum up, there might be a possible causal relationship between the topical use of brimonidine and acute uveal effusion in our case. Multiple factors took part in the onset of UES in our case: the anatomic changes due to nanophthalmos,YAG laser treatment, taking the squatting position, and most importantly, the topical use of brimonidine. More cases or studies in nanophthalmice eyes or animal models are necessary to demonstrate this relationship. It would be of interest to know if there are any measures that can prevent the onset of UES in nanophthalmic eyes, especially when asymptomatic idiopathic uveal effusion existed. We suggest paying attention to the clinical feature of nanophthalmos to avoid a misdiagnosis. UBM test evaluating the status of the ciliary body before prophylactic laser treatment is recommended to exclude the possibility of asymptomatic uveal effusion in nanophthalmic eyes. Topical brimonidine should be used with caution in nanophthalmic eyes.

## Data Availability

The datasets used and/or analyzed during the current case report are available from the corresponding author on reasonable request.

## Abbreviations

UES: Uveal effusion syndrome
PAC: Primary angle closure
UBM: Ultrasound biomicroscopy
IOP: Intraocular pressure
CT: Corneal thickness
AL: Axial length
OCT: Optical coherence tomography
